# The influence of social support on risk of acute cardiovascular diseases in female population aged 25–64 in Russia

**DOI:** 10.3402/ijch.v72i0.21210

**Published:** 2013-08-05

**Authors:** Valery V. Gafarov, Dmitry O. Panov, Elena A. Gromova, Igor V. Gagulin, Almira V. Gafarova

**Affiliations:** 1 Laboratory of Psychological and Sociological Issues of Internal Medicine, Institute of Internal Medicine, Siberian Branch of the Russian Academy of Medical Sciences, Novosibirsk, Russia; 2 Collaborative Laboratory of Epidemiology Cardiovascular Diseases, Siberian Branch of the Russian Academy of Medical Sciences, Novosibirsk, Russia

**Keywords:** social support, self-rated health, awareness, relative risk, myocardial infarction, stroke

## Abstract

**Objective:**

To study the prevalence of social support (SS) and its influence on the relative risk (RR) of myocardial infarction (MI) and stroke in the female population aged 25–64 in Russia.

**Materials and methods:**

Under the third screening of the WHO “MONICA-psychosocial” programme, a random representative sample of women aged 25–64 (n=870) were surveyed in Novosibirsk. SS was measured according to the methods of the Berkman–Sym test [indices of close contacts (ICC) and index of social network (SNI)]. From 1995 to 2010, women were followed for 16 years to observe the incidence of MI and stroke.

**Results:**

The prevalence of low levels of ICC and SNI in women aged 25–64 was 57.1 and 77.7%, respectively. Low levels of ICC and SNI were associated with poor self-rated health and awareness about their health, adverse behavioral habits, high job strain and family stress.

Rates of MI and stroke development were higher in married women with low ICC and SNI who were being in class “hard manual work”. Over a 16-year study period, the RR of MI in women with low ICC compared to those with high ICC was 4.9 times higher, and the risk of stroke was 4.1 times higher. Low level of SNI increased MI risk in 2.9 times, risk of stroke in 2.7 times.

**Conclusion:**

Majority of women aged 25–64 years in Russia have low social support which is associated with poor self-rated health, low awareness about the health that increases the risk of MI and stroke in 2.7–4.9 times in groups of “married” and “hard physical work”.

Recent studies have shown that low social support (SS) in the general population is more common in women than in men, and a small index of social network (SNI) in women is associated with coronary heart disease risk factors and coronary artery stenosis (1,2). Social isolation is associated with increased cardiovascular morbidity and all-cause mortality (3–6). Women are more likely to need social affiliation than men, with social deprivation and loss of SS being associated with greater sclerosis of coronary vessels for them (7–9).

The absence of such surveys in Russia necessitated a study on the prevalence and influence of SS (low levels of close contacts and SNI) on the relative risk (RR) of myocardial infarction (MI) and stroke over a period of 16 years, focussing on the relationship between SS and awareness, and attitude to health in the female population aged 25–64 in Russia/Western Siberia (Novosibirsk).

## Materials and methods

Within the framework of the third screening (1994) of the WHO programme “Multinational Monitoring of Trends and Determinants of Cardiovascular Disease” (MONICA) and sub-programme “MONICA-psychosocial (MOPSY)” (10), a random representative sample of women aged 25–64 (n=870) from one of Novosibirsk's districts were surveyed. The representative sample was generated on the basis of the electoral lists of citizens using a table of random numbers. The response was 72.5%.

This survey was performed using the standard methods accepted in the “MONICA study” protocol. The programme of psycho-social screening examinations includes registering of social characteristics such as marital status, level of degree, professional class and psychosocial tests.

SS was investigated at the baseline examination using a Berkman–Sym test (11); an index of close contacts (ICC) and index of SNI were measured. ICC level was assessed as high, average, low; SNI-high, average-1, average-2, low.

Questionnaire entitled “Awareness and attitude towards the health” was used for assessment. It covered attitude towards health and cardiovascular diseases (CVD) prevention; attitude towards smoking, diet and physical training; job and family stress.

Over a follow-up period (1995–2010), there were 35 cases (6.3%) of stroke incidence which were registered by means of examination, analysis of medical histories, cards and death certificates. A total of 15 cases (2.7%) of MI were revealed in the studied cohort using the WHO programme “Registry of Myocardial Infarction” data (12). Statistical processing was fulfilled by means of programme pack SPSS version 11.5. Cox-proportional regression model was used to estimate RR, taking into account different time intervals. Persons having baseline MI, and a history of stroke or heart disease and diabetes mellitus were excluded from analysis. The Chi-square test was used to test the statistical significance of differences between groups (χ^2^). Values of p<0.05 were considered statistically significant.

## Results

ICC levels in the female population aged 25–64 in 1994 were: a low level of index close contacts – 57.1%, average ICC – 37.3%, high – 5.7%. Prevalence of low levels of SNI (combined SNI-low, SNI-average 1) was 77.7%, SNI-average 2–19.8%, SNI-high – 2.5%.

There was a substantial growth of negative self-assessments health as “ill” and there was a reducing in assessments as “healthy” in women with low ICC (ICC-low – 14.5% and 11.2%; ICC-high – 7.1% and 17.9%, respectively); no differences were found for SNI index towards self-rated health. The trend of increasing complaints about health was evident in those with lowest SS (ICC-low – 90.1%; SNI-low – 90.3%). In conditions of low SS, women believe that taking care of their health is not enough (χ^2^=19.37, df=6, p<0.01) and note the high “probability to be ill in the next 5–10 years” (ICC-low – 59.2%, χ^2^=12.23, df=4, p<0.05; SNI-low – 57.4%, χ^2^=20.11, df=6, p<0.01).

Women with low ICC and SNI are more likely to stop working and seek medical help when they feel bad at work (χ^2^=12.76, df=6, p<0.05). However, 44% of those with low SNI do not consider influenza or febrility as a barrier and continue to work.

The following tendencies are likely in women with low ICC and SNI with regard to job stress: they are less likely to change their specialty, perform less additional tasks, dislike their work, assess their responsibility as “insignificant” and have less opportunity to relax after a typical working day. There was a drop in their capacity to work during the current year (χ^2^=17.43, df=9, p<0.05).

With increasing levels of S, there was stability in their marital status (ICC-low – 17.9%, ICC-high – 3.7%; SNI-low – 15.8%, SNI-high – 0%); conflicts in the family were on the rise (ICC-low – 55.6%, ICC-high – 67.9%, χ^2^=13.66, df=6, p<0.05; SNI-low – 57.3%, SNI-high – 78.6%; p>0.05).

With regard to smoking, women with low ICC and SNI have reduced motivation cut down smoking (answer “smoked less than a year ago”: ICC-low – 19.8%, ICC-high – 28.6%; SNI-low – 25%, SNI-high – 100%); they also try to follow a diet plan (ICC-low – 23%, ICC-high – 29.6%; SNI-low – 21.4%, SNI-high – 42.9%). But, such persons are 3 times less likely to respond that “do physical exercises regularly”; it is associated with a decline in the proportion of people spending their leisure productively (ICC-low – 19.9%, ICC-high – 35.6%, p>0.05; SNI-low – 23.6%, SNI-high – 28.6%, χ^2^=16.93, df=6, p<0.05).

The structure of marital status in a cohort of women with MI and low SS was as follows: married – 66.6 and 57.1%; divorced – 16.7 and 28.6%; widowed – 16.7 and 14.3% for low ICC and SNI, respectively. There were no unmarried women. There was a tendency of increased MI incidence in married women with low ICC and SNI indices compared to higher levels of SS.

The structure of marital status in a cohort of women with stroke and low SS was: never been married – 15.8 and 13%; married – 78.9 and 73.9%; divorced – 5.3 and 8.7%; widowed – 0 and 4.3% for low ICC and SNI, respectively. The incidence of stroke among married women with low ICC was higher in comparison with higher ICC (χ^2^=3.95, df=1, p<0.05). There was a tendency towards higher stroke incidence in married women with low SNI.

The educational level of persons with stroke and low ICC was: higher education (university) – 40%; incomplete higher/vocational education – 40%; elementary school – 20%. The share of women with MI and low SNI was equally distributed among the different grades of education – 33.3%. Women with higher and vocational education and low ICC are more likely to have higher rates of MI.

The structure of education in women with developed stroke and low ICC was as follows: higher education – 10.5 and 13%; incomplete higher/vocational education – 36.8 and 43.5%; high school – 31.6 and 26.1%; elementary school – 21.1 and 17.4% for low ICC and SNI, respectively. There was a tendency towards higher stroke rates in those with incomplete higher/vocational education and low ICC/SNI.

Professional status in women with MI and low ICC was presented in the following categories: 40% − heads, 20% − engineers, 40% − pensioners. In comparison, the structure in women with MI and low SNI had several differences: 33.3% − heads, 16.7% − engineers, 50% − pensioners. There was a tendency towards an increase of MI incidence in women who were a “head” having low ICC and SNI compared to high SS levels.

Professional status in women with stroke and low ICC was: 5.3% − middle executives, 15.8% – heads (managers), 10.5% − engineers, 15.8% – working hard and light physical labour, 21.1% – moderate physical labour, 5.3% − pensioners, 10.5% – military servants. The structure of professional status in group with developed stroke and low SNI was: 8.7% − middle executives, managers, engineers and working hard and light physical labour had an equal share to 13%, 21.7% – moderate physical labour, pensioners and military servants were equal shares − 8.7%. The rate of stroke incidence in people engaged in hard physical labour with low ICC was higher than in engineers (χ^2^=6.16, df=1, p<0.05) and pensioners (χ^2^=12.99, df=1, p<0.001) with low ICC. A significant increase in the rate of stroke was found in people engaged in severe physical labour with low SNI compared to pensioners with low SNI (χ^2^=7.72, df=1, p<0.01).

RR of MI in women aged 25–64 with a low ICC was 4.9 times higher (95% CI=1.108–21.762; p<0.05) compared to those with higher ICC levels; it was 4.1 times higher for stroke (95% CI=1.193–14.055; p<0.05) than in those with a higher level of ICC over the 16 years. Women with low SNI aged 25–64 had a 2.92-fold risk of MI incidence (95% CI=1.040–8.208; p<0.05) and a 2.72-fold risk of stroke (95% CI=1.094–6.763; p<0.05) compared to persons with a higher level of SNI. The risk of MI was substantially increased in the 55–64 age group and it was 5.9 times higher (95% CI=1.534–22.947; p=0.01) for women with lower SNI.

## Discussion

Our studies showed that self-rated health has deteriorated among the female population and that the number of health complaints has increased with decreasing SS. There is a lack of awareness of the female population about their health, which is consistent with other studies (13,14). Awareness about health is known to be related to the frequency of contact with family, friends and near and dear ones (15).

High levels of job and family stress were identified in women with low ICC and SNI levels. It is known that family stress in women of working age with ischemic heart disease affects their SNI, that is, it reduces social integration, sense of affiliation and tangible support (16).

In our study, low levels of ICC and SNI are associated with negative behavioral habits and the lack of any changes (low smoking cessation, physical activity, adherence to diet) are confirmed by other researchers (17). There is a necessity for well-developed SNI, which comes from the fact that high levels of SS can reduce the impact of negative effects and psycho-social factors on adverse lifestyle (smoking, alcohol, poor diet) (18).

Our findings show a significant effect of low levels of SS on the relative risk of MI and stroke in the female population of working age in Russia/Siberia. These data are confirmed by other longitudinal studies where low SS is a predictor of morbidity and mortality from acute CVD (19–21).

Based on our results, we observed that married women with low ICC and SNI indices are more likely to develop stroke and there is a tendency towards an increased MI incidence rate. This is probably due to higher levels of family stress in this group compared to the divorced and widowed group, and this is the confirmed opinion of other authors (22,23).

There was a significant increase in the incidence rate of stroke in the hard physical labour group in women with low levels of ICC and SNI. There is a tendency for an elevated MI incidence rate in women-executives (managers). The presence of intrapersonal conflict (family or career) and high levels of stress and job strain as shown in other studies is associated with a higher incidence of CVD in this group of women (24,25).

## Conclusions

- Prevalence of low levels of SS (ICC and SNI) in female population aged 25–64 in Russia/Siberia is high, 57.1 and 77.7%, respectively.
- Low levels of SS (ICC and SNI) associated with poor self-rated health, low level of awareness about the health, adverse behavioral habits in the female population aged 25–64 in Russia/Siberia.
- Over 16-year study period in the female population aged 25–64 in Russia/Siberia, low levels of SS (ICC and SNI) significantly increased the relative risk of MI and stroke, especially in the older age group.
- Low levels of SS (ICC and SNI) which lead to the development of MI and stroke in Russian (Siberian) women aged 25–64 associated with marital status “married”; average levels of a degree; professional status “the head”, “physical labourer”; high levels of family and job stress.
